# Proinflammatory Cytokines Increase Vascular Endothelial Growth Factor Expression in Alveolar Epithelial Cells

**DOI:** 10.1155/2015/387842

**Published:** 2015-09-03

**Authors:** James P. Maloney, Li Gao

**Affiliations:** ^1^Division of Pulmonary and Critical Care Medicine, University of Colorado Denver, 12700 East 19th Avenue, C272, Aurora, CO 80045, USA; ^2^Division of Allergy and Clinical Immunology, Department of Medicine, Johns Hopkins University, Room 3A.62A, 5501 Hopkins Bayview Circle, Baltimore, MD 21224-6801, USA

## Abstract

Vascular endothelial growth factor (VEGF) is an endothelial permeability mediator that is highly expressed in lung epithelium. In nonlung cells proinflammatory cytokines have been shown to increase VEGF expression, but their effects on lung epithelium remain unclear. We hypothesized that increases in alveolar epithelial cell VEGF RNA and protein expression occur after exposure to proinflammatory cytokines. We tested this using human alveolar epithelial cells (A549) stimulated with 5 proinflammatory cytokines. VEGF RNA expression was increased 1.4–2.7-fold in response to IL-1, IL-6, IL-8, TNF-*α*, or TGF-*β* over 6 hours, with TGF-*β* having the largest response. TNF-*α* increased VEGF RNA as early as 1 hour. A mix of IL-1, IL-6, and IL-8 had effects similar to IL-1. TNF-*α* increased protein expression as early as 4 hours and had a sustained effect at 16 hours, whereas IL-1 did not increase protein expression. Only VEGF_165_ was present in cultured A549 cells, yet other isoforms were seen in human lung tissue. Increased expression of VEGF in alveolar epithelial cells occurs in response to proinflammatory cytokines. Increased VEGF expression likely contributes to the pathogenesis of inflammatory lung diseases and to the angiogenic phenotype of lung cancer, a disease typically preceded by chronic inflammation.

## 1. Introduction

Acute and chronic inflammatory states are characterized by increased regional tissue concentrations of proinflammatory cytokines such as the interleukins IL-1, IL-6, and IL-8, transforming growth factor *β* (TGF-*β*), tumor necrosis factor *α* (TNF-*α*), and oxidant species [1]. Examples of the pathologic role of these cytokines in chronic inflammatory injury include the liver inflammation of hepatitis C infection, joint inflammation in rheumatoid arthritis, and smoking-related airway inflammation in chronic obstructive pulmonary disease and lung cancer [2–4]. These mediators are also typically found in high concentrations in lung tissue during acute inflammatory diseases [5–7], where they are typically associated with worse outcomes [5, 8] and seen in multiple cells resident in the lung [9, 10]. Chronic lung inflammation due to smoking or diseases such as cystic fibrosis is also characterized by regional increases of these mediators [11, 12]. These cytokines are typically released by leukocytes and macrophages within the inflamed lung and have pleiotropic effects on resident tissues that often lead to cell injury and fibrosis [12, 13].

Vascular endothelial growth factor-A (VEGF-A, hereafter referred to as “VEGF”) is a potent, endothelial specific permeability mediator and mitogen that is highly expressed in a number of organs including the lung [14–16]. VEGF is a proinflammatory dimeric protein that enhances endothelial permeability* in vivo* at nanomolar concentrations. VEGF exists in three common isoforms corresponding to the number of amino acids in protein monomers generated by alternative RNA splicing (VEGF_121_, VEGF_165_, and VEGF_189_). VEGF signals primarily via receptors which are expressed predominantly on endothelial cells but have been described on some epithelial cells [17].

Proinflammatory cytokines like IL-1, IL-6, IL-8, TGF-*β*, and TNF-*α* variably increase VEGF expression, depending upon the dose, cell, or tissue type [18–21]. VEGF in turn induces a number of proinflammatory genes and promotes transendothelial migration of neutrophils; thus, VEGF secretion can augment neutrophil-mediated inflammatory responses [22, 23]. In the lung, VEGF protein is predominantly expressed in bronchial and alveolar epithelium [24], but migrating inflammatory cells such as neutrophils and platelets can also release VEGF [25, 26]. Understanding the responses of alveolar epithelium to proinflammatory cytokines is needed to better elucidate the pathophysiology of acute and chronic lung diseases, but to date only a few studies have evaluated the alveolar epithelial cell VEGF response to proinflammatory cytokines. These studies have yielded indeterminate results, presenting only qualitative data or evaluating a single proinflammatory cytokine's effects [27, 28]. With this background, we hypothesized that proinflammatory cytokines increase the synthesis of VEGF in alveolar epithelium. We tested this hypothesis* in vitro* using human alveolar cells exposed to multiple proinflammatory cytokines and then assessing VEGF RNA and protein expression.

## 2. Materials and Methods

General laboratory reagents and plastics were from Sigma (St. Louis, MO). All media and buffers were obtained from Fisher (Pittsburgh, PA). All cytokines (except for VEGF) were from R&D Systems (Minneapolis, MN).

### 2.1. Cell Culture

Human alveolar epithelial cell culture: A549 Cells (ATCC CCL-185) were obtained from American Type Culture Collection (Manassus, VA) and were grown at 37°C with 5% CO_2_ and 21% O_2_ in a humidified incubator. Growth medium was DMEM (F12 Ham's) with 10% FCS, 10 mM HEPES, penicillin 100 U/mL, streptomycin 100 *μ*g/mL, amphotericin 0.25 *μ*g/mL, and NaHCO_3_ 9 mM. Medium was changed every two days until cells were subconfluent. Prior to cytokine stimulation, cells were serum-starved for 16 hours in media with 1% BSA substituted for FCS. Cells were then treated with cytokines (or PBS vehicle) at listed final concentrations: IL-1*β* (10 ng/mL; “IL-1”), IL-6 (80 ng/mL), IL-8 (100 ng/mL), TNF-*α* (10 ng/mL), and TGF-*β*_1_ (1 ng/mL; “TGF-*β*”). A noncytokine, phorbol 12-myristate 13-acetate (PMA, 0.1 *μ*M) was used as a positive control (its activation of protein kinase C has been shown to increase VEGF expression) [16]. A mix of IL-1, IL-6, and IL-8 at these concentrations was also used to test for effects on RNA expression. Incubation times varied: RNA expression of all cytokines was studied at 6 hours; time courses for RNA expression (TNF-*α*, IL-1*β* only) were at 1, 2, 4, and 8 hours; time courses for protein expression (TNF-*α*, IL-1*β* only) were performed at 2, 4, 8, and 16 hours. Cells were washed with warm PBS before lysis and isolation of RNA and protein.

### 2.2. RNA Isolation

Cells were placed on ice after the addition of 1 mL of Trizol (Life Technologies, Grand Island, NY) and scraped from plates with a disposable rubber policeman. Total cellular RNA was isolated following manufacturer's instructions.

### 2.3. Northern Blotting

Northern blots were performed after the methods of Lehrach et al. [29]. Twenty *μ*g of total cellular RNA was loaded into wells of denaturing 1.2% agarose gels and run at 95 volts in 1x MOPS buffer; RNA Millenium markers (Life Technologies) and a known 4.0 kb pCRII plasmid linearized with* EcoRI* (Life Technologies; ^32^P-labeled) were loaded in lane 1 or 10. Integrity of 18S and 28S RNA was verified with ethidium bromide staining before transfer. Gels were washed in 10x SSC, followed by RNA transfer to Zeta-probe membranes (Biorad). RNA was UV cross-linked and membranes were blocked at 68°C with Pre-Hyb solution (Clontech, Mountain View, CA) in glass cylinders in a rotating hybridization oven (Sigma). Specific cDNA probes were created by RT-PCR of VEGF (202 bp) and *β*-actin (416 bp) from 1 *μ*g of RNA from human total lung RNA (Clontech) using a kit (Promega, Madison, WI) and primers (VEGF: Fw 5′-TCC AGG AGT ACC CTG ATG AG-3′, Rv 5′-ATT CAC ATT TGT TGT GCT GT-3′, corresponding to sequences within exons 3-4 (shared among VEGF isoforms); *β*-actin: Fw 5′-CCG TTT TCC GTA GGA CTC TCT TCTC-3′, Rv 5′-ACA GGG ATA GCA CAG CCT GGA TAG-3′) followed by subcloning of gel-purified products into a TA subcloning kit (Life Technologies) and amplification with a maxi-prep kit (Qiagen, Valencia, CA). We verified inserts by DNA sequencing; then cDNA probes were excised with* EcoRI* and purified in spin columns. The dsDNA probes were radiolabeled with 2 *μ*Curie of *α* ^32^P-dCTP (GE Healthcare Life Sciences, Pittsburgh, PA) using DNA labeling beads (GE) and then purified with G-50 Sephadex spin columns (Sigma). Radiolabeled probe (1 × 10^6^ cpm) was added to 10 mL of Express-Hybe (Clontech) solution and hybridized over membranes at 68°C for 2 hours. Membranes were washed at 68°C in 2x SSC (4 times) and similarly in 0.1x SSC and then placed in a cassette with intensifying screens at −80°C overnight. Images were captured with a phosphorimager (GE). Membranes were stripped in 0.1% SDS and reincubated with a *β*-actin probe. This technique can detect VEGF RNA at 5.5, 4.4, and 3.7 kb and *β*-actin RNA at 2.1 kb and *β*-actin at 1.9 kb.

### 2.4. Protein Isolation

Cells were lysed with 0.6 mL of ice-cold RIPA buffer (150 mM NaCl, 1.0% Triton-X 100, 0.5% sodium deoxycholate, 0.1% SDS, and 50 mM Tris; pH 8.0) with freshly added proteinase inhibitors (PMSF final 1 mM, leupeptin final 1 *μ*M). Cells were scraped free with disposable policemen and transferred to microfuge tubes. Proteinase inhibitors were again added and lysates were incubated for 60 minutes on ice. Lysates were centrifuged at 15,000 ×g for 20 minutes, and supernatants with soluble protein were frozen at −70°C after concentrations were measured using a Bradford kit (Biorad).

### 2.5. Immunoprecipitation of VEGF

Antibodies were obtained from Santa Cruz Biotechnology (Santa Cruz, CA). 200 *μ*g of total cellular proteins was precleared by adding 0.25 *μ*g of control rabbit IgG-agarose beads (sc-2345) at 4°C on a rotating platform for 30 minutes, followed by pelleting at 1000 ×g for 5 minutes at 4°C (for this and all subsequent centrifugations, 20 *μ*L of protein A/G agarose resin (sc-2003) was added to each tube to allow pellet visualization). Supernatants were removed and incubated with 10 *μ*g of polyclonal rabbit IgG anti-VEGF-agarose conjugate (sc-152AC) for 16 hours at 4°C on a rotating platform. Negative control incubations were performed using nonimmune rabbit IgG-agarose (sc-2027) for this last step. Pellets were collected after centrifugation at 1000 ×g for 5 minutes at 4°C, washed with 0.5 mL RIPA buffer, resuspended in 40 *μ*L of reducing Laemmli sample buffer, boiled for 5 minutes, then aliquoted, and frozen at −70°C.

### 2.6. Western Blotting

General methods are as published [26]. Ten *μ*L of reduced immunoprecipitates was loaded onto 15% polyacrylamide minigels alongside molecular weight markers and 25 ng of recombinant VEGF_165_ standard (gift of Genentech). After electrophoresis proteins were transferred onto nitrocellulose membranes. Membranes were washed, blocked, and probed with a 1 : 500 polyclonal rabbit anti-VEGF IgG (sc-507) and then donkey anti-rabbit IgG-HRP (sc-2313), then washed again and placed in ECL solution (GE), and exposed to X-ray film. VEGF_165_ bands were identified by migration at 21 kDa (monomer). Band densities were measured after scanning. Membranes were stripped using a commercial buffer and following manufacturer's guidelines (Restore, Life Technologies); membranes were then blocked and reprobed for human *β*-actin protein (sc-81178) using the same techniques and scanned for *β*-actin density to normalize for unequal protein loading. With this technique *β*-actin is detected at a size of 42 kD. As blots showed presence of only the VEGF_165_ isoform, experiments were done with 10 ug of human lung protein (Clontech) to see if our technique could identify other isoforms previously detected in lung [15].

### 2.7. Statistical Analysis

Data are presented as mean ± SEM of at least 3–5 experiments per condition. Band densities were measured using NIH ImageJ software (NIH, Bethesda, MD); for northern blots and for quantitative western blots band densities were normalized to *β*-actin. Comparisons were performed using GraphPad software (GraphPad Inc., San Diego, CA). Intergroup differences were evaluated nonparametrically with one-sided ANOVA, Kruskal-Wallis for >2 group comparisons, or Mann-Whitney for two groups. Significance was defined as *P* < .05.

## 3. Results

### 3.1. VEGF RNA Is Constitutively Expressed in Human Alveolar Epithelial A549 Cells and Is Upregulated by Proinflammatory Cytokines IL-1, IL-6, IL-8, TNF-*α*, and TGF-*β*

**Figure 1:**
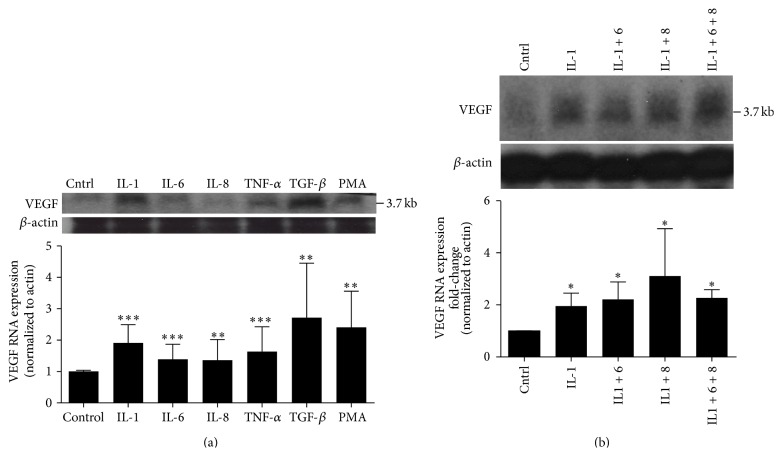
Northern blots showing VEGF RNA expression in A549 cells. Graphs below blots indicate fold-change in RNA expression compared to vehicle (Cntrl) and are normalized to *β*-actin expression. Phorbol ester (PMA) is included as a positive control in (a). Only a single VEGF band was seen at 3.7 kb, corresponding to the mRNA for VEGF_165_. (a) Results from 6 hours after stimulation with individual proinflammatory cytokines, showing VEGF bands with corresponding *β*-actin bands; graphic analysis of comparison to vehicle control (cntrl) is at the bottom of figure. (b) Results from 6 hours after stimulation with a mixture of IL-1, IL-6, and IL-8 compared to results with IL-1 alone, showing VEGF bands with corresponding *β*-actin bands. All conditions demonstrated increased VEGF expression compared to vehicle control (Cntrl), but effects of cytokine mixes are not significantly different than IL-1 alone, though trends are apparent. Graphic analysis is at the bottom of figure. Blots are representative of 6 individual experiments. ^*∗*^ *P* < .05, ^*∗∗*^ *P* < .01, and ^*∗∗∗*^ *P* < .001.

A549 cells displayed a prominent 3.7 kb band on northern blotting consistent with the known major band reported for VEGF RNA in most cells where VEGF is expressed (Figure 1(a)). Other bands were not seen. This band was present constitutively (in the absence of stimuli) but was more pronounced after stimulation with the positive control (PMA) and with 5 individual proinflammatory cytokines over 6 hours (Figure 1(a)). VEGF band densities were normalized to *β*-actin densities. Of the proinflammatory cytokines, TGF-*β* appeared to have the largest effect on upregulation of VEGF RNA, with an average response of a 2.72 ± 0.58 fold-change; this was actually more than the change of 2.41 ± 0.41-fold seen with the positive control PMA (mean ± SEM). The proinflammatory cytokine with the smallest effect was IL-8 (1.36 ± 0.27-fold-change) but this was also statistically higher than the control (vehicle) expression. A mix of stimulatory interleukins did not appear to have significant synergistic effects on VEGF RNA expression versus that of IL-1 alone (Figure 1(b)): IL-1, IL-6, and IL-8 together increased VEGF RNA 2.27 ± 0.16-fold (mean ± SEM) at 6 hours, whereas IL-1 increased VEGF RNA at a 1.95 ± 0.22-fold-change, and a mix of IL-1 + IL-8 had the greatest response (a 3.1 ± 0.91-fold-change). While there were trends for some cytokine mixes to have effects greater than that of IL-1 alone, at the number (*N*) of 4 in these experiments there was no statistically significant difference between the groups (Figure 1(b)). Of note, the individual effect of IL-1 was the greatest of the 3 interleukins tested in this manner (Figure 1(a)). The cDNA probe we used for northern blotting is complementary to sequences shared among all VEGF isoforms generated by alternative splicing (including the antiangiogenic “b” isoforms), so the presence of a single RNA band for VEGF in these experiments (in both stimulated and unstimulated conditions) suggests that the A549 cells we utilized produce only one VEGF isoform.

### 3.2. VEGF RNA Is Upregulated Early after Cytokine Stimulation in A549 Cells

**Figure 2:**
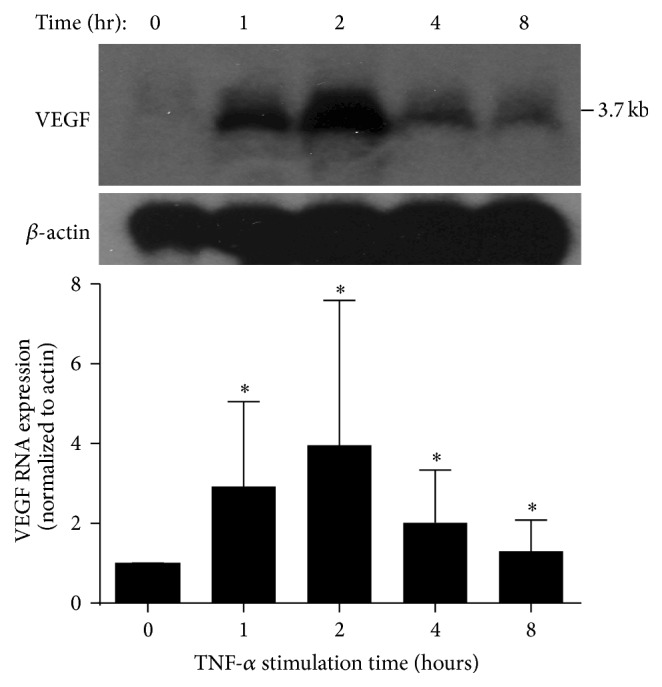
Northern blots showing effects over a time course of cytokines on VEGF RNA expression after stimulation with TNF-*α*. Results are expressed as fold-change compared to time zero, normalized to *β*-actin. Graphic analysis is at the bottom of the figure; all time points show increased expression versus time zero but do not differ from each other, though an apparent peak effect is suggested at 2 hours. Blots are representative of 4 individual experiments. ^*∗*^ *P* < .05.

In separate experiments, an increase in VEGF RNA expression was seen rapidly after proinflammatory cytokine stimulation, appearing as early as one hour after stimulation with TNF-*α*, with a peak effect at 4 hours (Figure 2). VEGF RNA remained minimally upregulated at 8 hours. Only TNF-*α* was tested as the hypothesis of these experiments (that VEGF upregulates very early in inflamed lung epithelium) was validated after testing of TNF-*α* alone.

### 3.3. VEGF_165_ Protein Is Constitutively Expressed in Human Alveolar Epithelial A549 Cells and Is Upregulated by TNF-*α*, but Not by IL-1

**Figure 3:**
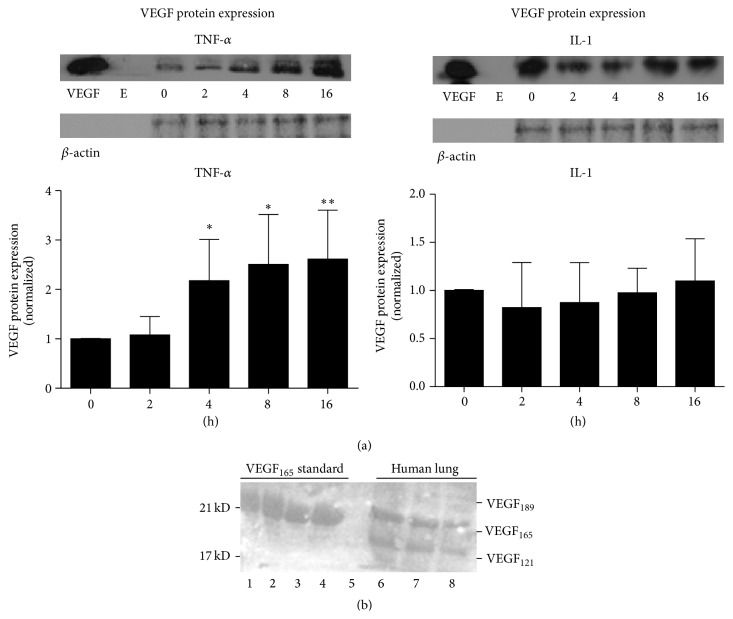
Western blots of VEGF protein expression. All proteins were reduced so that VEGF runs in monomeric form. (a) Time course (hours) of expression as fold-change after stimulation of cultured A549 cells with TNF-*α* and IL-1, showing increased protein expression of a single 21 kDa band corresponding to VEGF_165_ with TNF-*α* stimulation but not after IL-1 stimulation (compared to time zero). A VEGF_165_ standard (10 ng) was loaded in the leftmost lane “VEGF” and the contiguous lane is empty “E” for each condition. Blots were stripped and reprobed for *β*-actin expression. Results are expressed as fold-change compared to time zero, normalized to *β*-actin. (b) A human lung homogenate contains the 3 common VEGF isoforms, although only VEGF_165_ and VEGF_121_ appear as prominent bands (lanes 6–8). Lanes 1–4 contain a recombinant VEGF_165_ standard, and lane 5 is empty. Molecular weight is denoted at left. All blots are representative of 5 experiments for each condition. ^*∗*^ *P* < .05, ^*∗∗*^ *P* < .01.

Having seen positive effects on upregulation of VEGF RNA with proinflammatory cytokines, we next studied the effects of two of these cytokines (TNF-*α* and IL-1) on VEGF protein expression within A549 cells. We studied total cellular protein content; we did not study protein secretion into media as this had been previously reported by others [27, 28, 30]. We picked this subset of cytokines as they are particularly relevant to the systemic inflammatory states that predispose to ALI. Using immunoprecipitation techniques and a time course that assessed effects up to 16 hours after stimulation, we found that TNF-*α* increased VEGF protein expression as early as 4 hours, with a peak effect at 16 hours yielding a 2.6 ± 0.5-fold-change (mean ± SEM) (Figure 3(a)). Despite having positive effects on VEGF RNA, we did not find an effect of IL-1 on VEGF protein expression within A549 cells (Figure 3(a)).

### 3.4. VEGF_121_ and VEGF_189_ Protein Isoforms Are Not Significantly Expressed in A549 Cells, Either Constitutively or upon Stimulation with Proinflammatory Cytokines

We found no evidence that human alveolar epithelial cells expressed protein for the 121 or 189 amino acid isoforms (Figure 3(a)), either constitutively or after stimulation with cytokines. This was not a limitation of our technique, as we could demonstrate the presence of the three common isoforms of VEGF in human lung tissue, as has been previously reported (Figure 3(b)) [31].

## 4. Discussion

We found that VEGF-A (hereafter referred to as “VEGF”) RNA and protein expression increased in human alveolar epithelial cells after stimulation with a panel of proinflammatory cytokines relevant to acute and chronic inflammation (IL-1, IL-6, IL-8, TNF-*α*, and TGF-*β*). Stimulatory effects on VEGF RNA expression occurred with all cytokines and displayed a rapid response, which was previously described for VEGF only in endothelial cells [32]. VEGF RNA expression increased with a mixture of three interleukins, but only as a trend compared to the effect of IL-1 alone. Increased RNA expression was paralleled by an early increase in VEGF protein expression after stimulation by TNF-*α* (significant at 4 hours), but not after stimulation by IL-1 (protein expression was not measured for other cytokines). We only found evidence for the expression of one VEGF isoform in alveolar epithelial cells (VEGF_165_), though our techniques could detect other common VEGF isoforms in a human lung homogenate.

Our data suggest that increased alveolar epithelial VEGF production and secretion during acute or chronic lung inflammation may contribute to a vicious cycle, given the endothelial permeability and proinflammatory effects of VEGF. The increase in VEGF expression induced by inflammatory cytokines in human lung epithelial cells has relevance to acute inflammatory diseases such as pneumonia and acute lung injury and to chronic inflammatory lung diseases such as cystic fibrosis and bronchogenic lung cancer [11]. In lung cancer, increased VEGF expression due to chronic inflammation induced by cigarette smoking may also foster angiogenesis and growth of tumors [33, 34].

Our systematic evaluation of the effects of multiple proinflammatory cytokines on VEGF RNA and protein expression in human alveolar epithelial cells is unique and adds significantly to prior work in the field of VEGF-mediated lung pathobiology. It is important to put our findings in the context of the work of others. Qualitative increases in VEGF RNA expression and protein secretion into media of IL-1 and TNF-*α* stimulated A549 cells were previously reported but no statistical analyses were performed to support these conclusions [27, 30]. Others actually reported decreased VEGF secretion into A549 cell media with H_2_0_2_ stimulation or after acid exposure [31], while hyperoxia did not change VEGF RNA levels [35, 36]. Boussat et al. reported no effect of TNF-*α* on A549 cell VEGF secretion into media but noted increased VEGF secretion after TGF-*β* stimulation [28]. Notably, exogenous VEGF appears to increase proliferation of cultured human primary type II alveolar cells, suggesting that epithelial cell VEGF may have paracrine effects that aid epithelial cell resurfacing in inflammatory lung diseases or alternatively could stimulate lung cancer cells [15, 31]. While we could find only RNA and protein for a single VEGF isoform (VEGF_165_) in A549 cells, others have found multiple VEGF isoforms in these [31, 36]. Notably, our northern blot probe and anti-VEGF antibody were targeted to areas shared by all VEGF isoforms, including the antiangiogenic “b” isoforms, which differ from the canonical VEGF isoforms by alternative splicing of exon 8 with size differences too small to detect by northern blotting [15].

Our work has limitations. We did not look at the VEGF protein response to all of the proinflammatory cytokines we studied; instead we focused on the effects of TNF-*α* and IL-1. Moreover, epithelial secretion of VEGF* in vivo* could be impaired in later stages of inflammatory lung disease due to widespread epithelial damage, explaining the decreases in VEGF in alveolar lining fluid and lung tissue seen in later stages of some inflammatory lung diseases [37, 38]. We examined the responses of only a single epithelial cell line. Lastly, we could have done quantitative RT-PCR with primers specific to canonical VEGF isoforms and “b” isoforms in order to assess if their responses to proinflammatory cytokines were similar or discordant.

## 5. Conclusions

Human lung alveolar epithelial cells upregulate VEGF RNA and protein in response to proinflammatory cytokines. These data suggest that resident alveolar epithelial cells are an important source of lung VEGF in acute and chronic lung inflammatory disorders.

## Abbreviations

A549: Human alveolar epithelial cell line
ALI: Acute lung injury
IL-1, IL-6, or IL-8: Interleukins 1, 6, and 8
kDa: Kilodalton
MW: Molecular weight
*N*: Number
NFDM: Nonfat dried milk
PMA: Phorbol 12-myristate 13-acetate
TGF-*β*: Transforming growth factor-*β*_1_
TNF-*α*: Tumor necrosis factor *α*
VEGF: Vascular endothelial growth factor
VEGF_121_, VEGF_165_, and VEGF_189_: Protein isoforms of VEGF where numbers denote amino acid lengths.
